# Survival after laparoscopy versus laparotomy for apparent early-stage uterine clear cell carcinoma: Results of a large multicenter cohort study

**DOI:** 10.3389/fonc.2022.975485

**Published:** 2022-09-05

**Authors:** Chengyu Shui, Lin Ran, Yong Tian, Li Qin, Xin Gu, Hui Xu, Cui Hu, Lin-Lin Zhang, You Xu, Chen Cheng, Wu Huan

**Affiliations:** ^1^ Department of Obstetrics and Gynecology, Central Hospital of Enshi Tujia and Miao Autonomous Prefecture, Enshi Clinical College of Wuhan University, Enshi, China; ^2^ Department of Obstetrics and Gynecology, Mianzhu City People’s Hospital, Mianzhu, China; ^3^ Department of Obstetrics and Gynecology, Sichuan University West China Hospital (Mianzhu Hospital), Mianzhu, China; ^4^ Department of Obstetrics and Gynecology, West China Second University Hospital, Sichuan University, Chengdu, China; ^5^ Department of Obstetrics and Gynecology, The Second Affiliated Hospital of Chengdu Medical College, Chengdu, China

**Keywords:** uterine clear cell carcinoma, laparoscopy, surgical staging, overall survival, disease-free survival

## Abstract

**Objective:**

To compare the long-term survival between laparoscopic surgery and open surgery in patients with apparent early-stage uterine clear cell carcinoma (UCCC).

**Patients and methods:**

254 patients with apparent early-stage UCCC were reviewed. Comparisons were made between patients who underwent laparoscopic surgery versus those who underwent open surgery. Baseline data, clinicopathological data, and oncological outcomes were analyzed. 5-year disease-free survival (DFS) rate and 5-year overall survival (OS) rate were estimated and compared using the Kaplan-Meier method and the Log-rank test. The Cox proportional hazard regression model was employed to control the confounding factors.

**Results:**

147 patients underwent laparoscopic surgery, and 107 patients were managed by open surgery. No differences in terms of recurrence rate (laparoscopy versus laparotomy: 10.9% versus 12.9%, *P*=0.842) and recurrence pattern were observed. For patients who underwent open surgery and patients who underwent laparoscopic surgery, the 5-year DFS rates and 5-year OS rate were 75.8% (95% CI: 65.8%-83.2%) and 69.1% (95% CI: 58.8%-77.4%), 66.0% (95% CI: 57.1%-73.5%) and 60.8% (95% CI: 52.0%-68.5%), respectively. The Cox proportional hazards regression model shown that for apparent early-stage UCCC, the approach of surgical staging was not an independent predictor for survival (laparoscopy versus laparotomy: for DFS, aHR=1.06, 95% CI=0.64-1.75, *P*=0.826; for OS, aHR=1.10, 95% CI=0.72-1.68, *P*=0.671).

**Conclusion:**

For apparent early-stage UCCC, in terms of oncological survival, laparoscopic surgery was as safe as open surgery.

## Introduction

Generally, endometrial cancer (EC) can be broadly divided into type I tumors (approximately 80%) and type II tumors (approximately 20%) (1–3). Usually developing among the elderly, Type II EC has a hormone-independent pathogenesis and no identified precursor lesions (1, 3, 4). Including uterine serous carcinoma, uterine clear cell carcinoma (UCCC), and carcinosarcoma, type II EC typically has a worse prognosis when compared with type I EC (1–3). They are often present at advanced stages, have a high rate of extrauterine metastases, and are at high risk of recurrence after initial management (1, 2, 4).

For clinical early-stage EC, the primary management is surgical staging, at least including total hysterectomy, bilateral salpingo-oophorectomy, and the assessment of regional lymph nodes (1, 3, 4). Based on the results of two randomized prospective studies comparing minimally invasive surgery with traditional open surgery, the minimally invasive approach was recommended for early-stage EC by the European Society of Gynaecological Oncology, the European Society for Radiotherapy and Oncology, and the European Society of Pathology (5). Furthermore, pooled results of prospective studies and retrospective observational studies also support the employment of minimally invasive surgery for women with high-risk early-stage EC (including type II EC) (6–10). These studies concluded that when compared with those who were managed with open surgical staging, early-stage EC patients who were treated with minimally invasive surgery experienced similar survival, quicker recovery, and lower risk of perioperative complications (5–7, 9, 10). In 2018, however, two clinical studies reported that for women with early-stage cervical cancer, minimally invasive radical hysterectomy caused lower rates of disease-free survival (DFS) and overall survival (OS) than open radical hysterectomy (11, 12). Since then, the oncological safety of minimally invasive surgery for gynecologic malignancies has once again become a focus of attention in clinical studies.

UCCC accounts for less than 10% of all EC (1, 13, 14). Due to the rarity of UCCC, a large, powerful, and prospectively designed study regarding the management of UCCC is exceedingly difficult (14). Thus, the current data on the clinical practice of UCCC are usually from small and retrospective designed studies (5, 7, 13). In the aforementioned studies comparing minimally invasive surgery with traditional open surgery for high-risk endometrial cancer, the fraction of UCCC was fairly low (6, 7, 9, 10). Thus, the oncological safety of minimally invasive surgery for clinical early-stage UCCC needs further study.

Taken together, based on four Chinese high-volume teaching hospitals, we conducted this study to compare the risk of recurrence and death associated with minimally invasive surgery versus open surgery for clinical early-stage UCCC.

## Patients and methods

### Study design

With four Chinese high-volume centers involved, this was a retrospectively designed and multi-institutional cohort study. Due to the retrospective nature and it did not report any identifiable private data, ethical approval and written informed consent for participation were not required for this study in accordance with the local legislation and institutional requirements. This study was conducted following the Declaration of Helsinki (15).

### Study cohort

Data of consecutive patients with histologically proven EC who underwent surgical staging at the four Chinese tertiary referral centers (Central Hospital of Enshi Tujia and Miao Autonomous Prefecture, West China Mianzhu Hospital, West China Second University Hospital, and the Second Affiliated Hospital of Chengdu Medical College) between January 1, 2011 and January 1, 2018 were reviewed. Patients were included in this study if they: (1) were between 18 and 75 years old, (2) had pathologically confirmed clear cell carcinoma, (3) had a clinical early-stage disease, (4) underwent comprehensive surgical staging at the participating hospitals, at least including total hysterectomy, bilateral salpingo-oophorectomy, and pelvic lymphadenectomy, and (5) were consecutively followed up at these hospitals. In the current study, the clinical early-stage disease was defined as follows: cancer clinically confined to the uterus, no clinical evidence of bulky lymph nodes, and no clinical evidence of extrauterine macroscopic lesions. After surgical staging, all included cases were staged using the 2009 International Federation of Gynecology and Obstetrics (FIGO) staging system for EC.

Patients were excluded from this study if they: (1) were non-surgically managed, (2) underwent neoadjuvant therapies, (3) had a suspected advanced disease, (4) had synchronous cancer(s), (5) had a history of malignancy of the female reproductive system, (6) had a preoperative American Society of Anesthesiologists (ASA) physical status score of larger than III, (7) underwent assessment for regional lymph nodes by sentinel lymph node mapping, or (8) were lost to follow-up.

### Data collection

The collected data regarding clinicopathological characteristics were as follows: year of diagnosis, age at diagnosis, marital status at diagnosis, body mass index (BMI) at diagnosis, the preoperative ASA physical status score, the stage of disease (based on the 2009 FIGO staging system), the grade of tumor differentiation, the size of the primary tumor, whether there was lymphovascular space invasion (LVSI), and the result of peritoneal cytology.

The following data on treatment were collected: the approach of surgical staging (laparoscopy or laparotomy), the scope of regional lymphadenectomy (pelvic lymphadenectomy or combined pelvic and para-aortic lymphadenectomy), the protocol of postoperative adjuvant therapies (chemotherapy, radiation, or chemoradiation).

The following data regarding oncological outcomes were collected: the vital status of the patient, disease recurrence (site and date), date of death, and the cause of death. In this study, all included patients were followed up until death or January 1, 2022.

### Outcomes of interest

In the current study, the 5-year DFS rate and the 5-year OS rate were the primary outcomes of interest. DFS was defined as the time between the date of surgical staging for UCCC and the date of documented disease recurrence or death contributed by UCCC. OS was defined as the time from the date of surgical staging for UCCC to the date of documented death caused by any cause.

In this study, the secondary outcomes of interest were the independent predictors for the long-term survival of women with clinical early-stage UCCC.

### Statistical analysis

Statistical analyses were performed using IBM SPSS version 25 (SPSS Inc., Chicago, IL, USA). The Kaplan-Meier survival curves were generated by Stata version 17 (Stata Corp., College Station, TX, USA).

Based on the type of surgical staging approach, the included cases were divided into the laparoscopy group and the laparotomy group. Data on the characteristics of the study cohort were reported using standard descriptive statistics. Comparisons were made between the two groups using the chi-squared test or Fisher exact test for categorical variables and the *t*-test or the Wilcoxon rank-sum test for continuous variables. The 5-year DFS rate and the 5-year OS rate of the two groups were estimated and compared using the Kaplan-Meier method and the log-rank test. Hazard ratio (HR) and 95% confidence interval (CI) were calculated. The Cox proportional hazard regression model was employed to control the confounding factors. Candidate variables that were with a *P* value of less than 0.05 on univariate analysis or that were considered clinically relevant were included in the Cox proportional hazard regression model.

In the study, A two-sided *P* value of less than 0.05 was considered statistically significant.

## Results

### Characteristics of the study cohort

**Figure 1:**
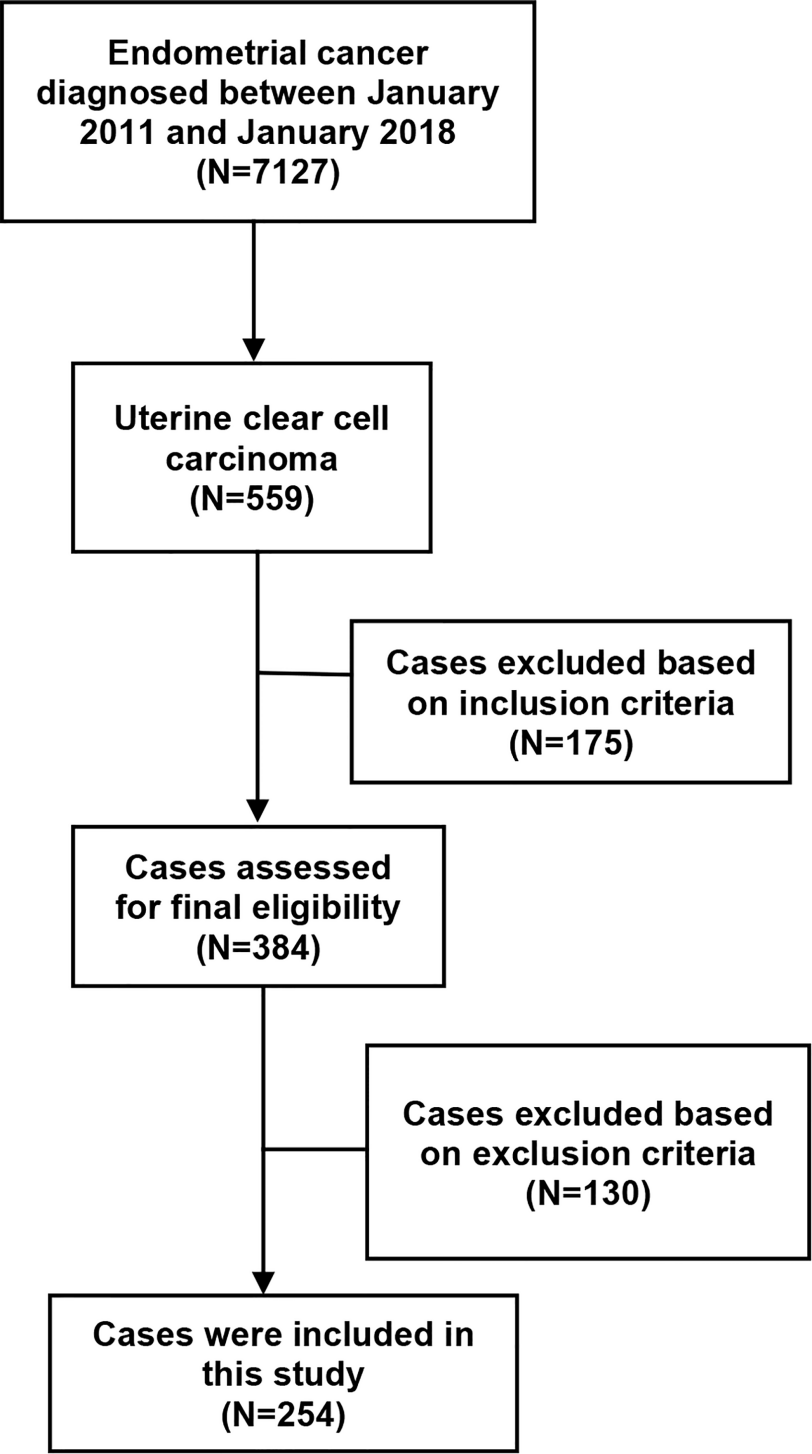
The flowchart of case selection.

A total of 7127 women with EC were diagnosed and managed at these four participating hospitals between January 1, 2011 and January 1, 2018. After excluding 6873 patients who were not eligible for this study, a total of 254 women with apparent early-stage UCCC were eventually included in the current study. Among them, 147 patients underwent surgical staging by laparoscopy and were included in the laparoscopy group, the remaining 107 women underwent surgical staging by open approach and were included in the laparotomy group. **Figure 1** shows the process of case selection.

For the entire study cohort, the mean age at diagnosis was 65.5 years with a standard deviation of 6.57, and the median duration of follow-up was 52.0 months (range: 4.0-131). Among the entire study cohort, 76 (29.9%) patients were identified with advanced diseases after surgical staging, 122 (48.0%) patients had primary tumors of larger than 4 centimeters, 58 (22.8%) patients were identified with LVSI, 36 (14.2%) patients had positive peritoneal cytology, and only 87 (34.3%) patients did not undergo any form of postoperative adjuvant therapy. In terms of the surgical-pathological stage of the disease, there was no statistical difference between the two groups (*P*=0.158).

**Table 1** shows the comparisons of the characteristics of the two groups. Generally, there was good comparability between the laparoscopy group and the laparotomy group in terms of the baseline characteristics, the clinicopathologic data, and the treatment-related variables.

**Table 1:** Characteristics of the study cohort^a^.

	Overall (N=254)	The laparoscopy group (N=147)	The laparotomy group (N=107)	*P*
Years of diagnosis				0.373
2011-2014	113 (44.5%)	69 (46.9%)	44 (41.1%)	
2015-2018	141 (55.5%)	78 (53.1%)	63 (58.9%)	
Age at diagnosis	65.5 ± 6.57	65.7 ± 6.46	65.3 ± 6.74	0.622
Duration of follow-up	52.0 (4.00, 131)	53.0 (4.00, 131)	49.0 (4.00, 127)	0.838
Marital status				0.612
Married	130 (51.2%)	73 (49.7%)	57 (53.3%)	
Single^b^	124 (48.8%)	74 (50.3%)	50 (46.7%)	
Body Mass Index^c^	21.2 ± 4.57	21.1 ± 4.30	21.3 ± 4.94	0.733
ASA physical status score				0.422
I/II	168 (66.1%)	94 (63.9%)	74 (69.2%)	
III	86 (33.9%)	53 (36.1%)	33 (30.8%)	
2009 FIGO stage				0.158
I	153 (60.2%)	82 (55.8%)	71 (66.4%)	
II	25 (9.8%)	13 (8.8%)	12 (11.2%)	
III	57 (22.4%)	38 (25.9%)	19 (17.8%)	
IV	19 (7.5%)	14 (9.5%)	5 (4.7%)	
Grade				0.687
Poorly differentiated	170 (66.9%)	100 (68.0%)	70 (65.4%)	
Undifferentiated	84 (33.1%)	47 (32.0%)	37 (34.6%)	
Tumor size				0.309
< 4 cm	132 (52.0%)	72 (49.0%)	60 (56.1%)	
≥ 4 cm	122 (48.0%)	75 (51.0%)	47 (43.9%)	
LVSI				0.762
No	196 (77.2%)	112 (76.2%)	84 (78.5%)	
Yes	58 (22.8%)	35 (23.8%)	23 (21.5%)	
Peritoneal cytology				0.147
Negative	218 (85.8%)	122 (83.0%)	96 (89.7%)	
Positive	36 (14.2%)	25 (17.0%)	11 (10.3%)	
Lymphadenectomy				0.429
Pelvic	162 (63.8%)	97 (66.0%)	65 (60.7%)	
Pelvic and para-aortic	92 (36.2%)	50 (34.0%)	42 (39.3%)	
Adjuvant therapy				0.961
CT or RT	96 (37.8%)	56 (38.1%)	40 (37.4%)	
CT plus RT	71 (28.0%)	40 (27.2%)	31 (29.0%)	
No	87 (34.3%)	51 (34.7%)	36 (33.6%)	

a Values are presented as mean ± standard deviation, median (minimum–maximum), or as number (percentage).

b Including never married, widowed, divorced, separated.

c Calculated as weight in kilograms divided by the square of height in meters.

ASA, American Society of Anesthesiologists; CT, Chemotherapy; FIGO, the International Federation of Gynecology and Obstetrics; LVSI, Lymphovascular Space Invasion; RT, Radiotherapy.

### Rates and patterns of recurrence

By January 1, 2022, 29 recurrences of UCCC were identified, and the rate of recurrence was 11.4% among the entire study cohort.

13 of the 107 patients (12.1%) in the laparotomy group had disease recurrence, and 16 cases of UCCC recurrence (10.9%) were identified in the laparoscopy group. In terms of the rate of disease recurrence, there was no statistical difference observed between the two groups (*P*=0.842). As for the patterns of disease recurrence, the most four common sites of recurrence were the abdomen (2.8%), the pelvis (2.4%), the lung (2.4%), and the vagina (1.6%). Also, there was no statistical difference observed between the two groups in terms of the patterns of disease recurrence. **Table 2** presents the rates and the patterns of recurrence by laparoscopic surgery versus laparotomy.

**Table 2:** Rates and patterns of disease recurrence^a^.

	Overall (N=254)	The laparoscopy group (N=147)	The laparotomy group (N=107)	*P*
Recurrence				0.842
Yes	29 (11.4%)	16 (10.9%)	13 (12.1%)	
No	225 (88.6%)	131 (89.1%)	94 (87.9%)	
Site of recurrence				
Vagina	4 (1.6%)	2 (1.4%)	2 (1.9%)	> 0.999
Pelvis	6 (2.4%)	3 (2.0%)	3 (2.8%)	0.699
Abdomen	7 (2.8%)	5 (3.4%)	2 (1.9%)	0.702
Nodal	2 (0.8%)	1 (0.7%)	1 (0.9%)	> 0.999
Lung	6 (2.4%)	3 (2.0%)	3 (2.8%)	0.699
Bone	2 (0.8%)	1 (0.7%)	1 (0.9%)	> 0.999
Multiple	2 (0.8%)	1 (0.7%)	1 (0.9%)	> 0.999

a Values are presented as number (percentage).

### Survival outcomes

For the patients who underwent surgical staging by open surgery and the patients who underwent laparoscopic surgery, the 5-year DFS rates by the Kaplan-Meier method were 75.8% (95% CI: 65.8%-83.2%) and 66.0% (95% CI: 57.1%-73.5%), respectively. For patients of apparent early-stage UCCC, surgical staging by laparoscopy was not associated with worse DFS when compared with traditional laparotomy (HR: 1.34, 95% CI: 0.85-2.11, *P*=0.213).

For the laparotomy group, the 5-year OS rate by the Kaplan-Meier method was 69.1% (95% CI: 58.8%-77.4%). Similarly, the 5-year OS rate for patients in the laparoscopy group was 60.8% (95% CI: 52.0%-68.5%). The comparison made by the Log-rank test indicated that for women with clinical early-stage UCCC, compared with open surgery, surgical staging by laparoscopy did not increase the risk of all-cause death (HR: 1.19, 95% CI: 0.81-1.76, *P*=0.372).

**Figure 2:**
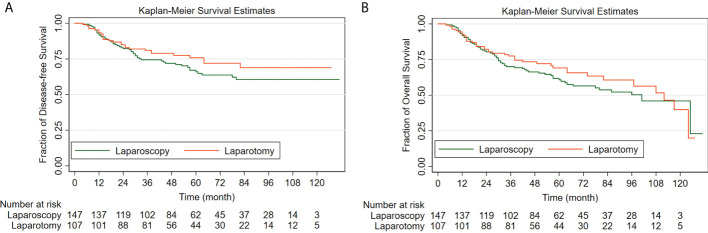
The Kaplan-Meier survival curves of the study cohort by laparoscopy versus laparotomy **(A)** for disease-free survival; **(B)** for overall survival.

**Figure 2** shows the Kaplan-Meier survival curves of the study cohort by laparoscopy versus laparotomy, **Figure 2A** for disease-free survival and **Figure 2B** for overall survival.

### Univariate analyses

Using the log-rank test, we found that for women with apparent early-stage UCCC, age at diagnosis (≥ 65 years versus < 65 years: for DFS, HR=1.66, 95% CI=1.07-2.77, *P*=0.031; for OS, HR=1.84, 95% CI=1.18-2.88, *P*=0.007), BMI at diagnosis (≥ 24 kg/m^2^ versus < 24 kg/m^2^: for DFS, HR=1.52, 95% CI=1.17-2.29, *P*=0.019; for OS, HR=1.46, 95% CI=1.09-2.14, *P*=0.037), the preoperative ASA physical status score (III versus I/II: for DFS, HR=3.51, 95% CI=2.14-4.99, *P*=0.000; for OS, HR=3.25, 95% CI=1.89-5.28, *P*=0.000), the 2009 FIGO stage of the disease (III/IV versus I/II: for DFS, HR=6.34, 95% CI=4.43-8.30, *P*=0.000; for OS, HR=5.95, 95% CI=3.38-10.01, *P*=0.007), the tumor size (≥ 4 cm versus < 4 cm: for DFS, HR=2.05, 95% CI=1.29-3.24, *P*=0.002; for OS, HR=1.80, 95% CI=1.22-2.64, *P*=0.003), LVSI (Yes versus No: for DFS, HR=1.54, 95% CI=1.04-2.70, *P*=0.013; for OS, HR=1.43, 95% CI=1.17-2.36, *P*=0.015), and postoperative adjuvant therapy (chemotherapy/radiotherapy versus No: for DFS, HR=0.68, 95% CI=0.27-0.89, *P*=0.007; for OS, HR=0.62, 95% CI=0.23-0.92, *P*=0.008; combined chemotherapy and radiotherapy versus No: for DFS, HR=0.45, 95% CI=0.29-0.87, *P*=0.004; for OS, HR=0.37, 95% CI=0.25-0.73, *P*=0.000) were associated with the prognosis.

**Table 3** shows the results of the univariate analyses.

**Table 3:** Univariate analyses of survival for apparent early-stage uterine clear cell carcinoma.

	OS			DFS		
	HR	95% CI	*P*	HR	95% CI	*P*
Age at diagnosis						
< 65 years	1			1		
≥ 65 years	1.84	1.18-2.88	0.007	1.66	1.07-2.77	0.031
Marital status						
Married	1			1		
Single	1.08	0.68-1.72	0.736	1.13	0.65-1.96	0.664
BMI at diagnosis						
< 24 kg/m^2^	1			1		
≥ 24 kg/m^2^	1.46	1.09-2.14	0.037	1.52	1.17-2.29	0.019
ASA physical status score						
I/II	1			1		
III	3.25	1.89-5.28	0.000	3.51	2.14-4.99	0.000
2009 FIGO stage						
I/II	1			1		
III/IV	5.95	3.38-10.01	0.007	6.34	4.43-8.30	0.000
Grade						
Poorly differentiated	1			1		
Undifferentiated	1.12	0.74-1.68	0.600	1.34	0.81-2.21	0.258
Tumor size						
< 4 cm	1			1		
≥ 4 cm	1.80	1.22-2.64	0.003	2.05	1.29-3.24	0.002
LVSI						
No	1			1		
Yes	1.43	1.17-2.36	0.015	1.54	1.04-2.70	0.013
Peritoneal cytology						
Negative	1			1		
Positive	1.12	0.71-1.77	0.622	1.11	0.66-1.89	0.692
Lymphadenectomy						
Pelvic	1			1		
Pelvic plus para-aortic	1.07	0.72-1.59	0.744	0.99	0.62-1.59	0.987
Adjuvant therapy						
No	1			1		
CT or RT	0.62	0.23-0.92	0.008	0.68	0.27-0.89	0.007
CT plus RT	0.37	0.25-0.73	0.000	0.45	0.29-0.87	0.004

ASA, American Society of Anesthesiologists; BMI, Body Mass Index; CI, Confidence Interval; CT, chemotherapy; DFS, Disease-free Survival; FIGO, the International Federation of Gynecology and Obstetrics; HR, Hazard Ratio; LVSI, Lymphovascular Space Invasion; OS, Overall Survival; RT, radiotherapy.

### Cox proportional hazards regression analyses

Variables that have potential clinical relevance or that showed a univariate relationship (*P* < 0.05) with survival were included in the multivariate Cox proportional hazards regression model, they were as follows: age at diagnosis, BMI at diagnosis, the preoperative ASA physical status score, the 2009 FIGO stage of the disease, the tumor size, the status of LVSI, the approach of surgical staging, and postoperative adjuvant therapy.

The Cox proportional hazards regression model showed that for apparent early-stage UCCC, the approach of surgical staging was not an independent predictor for long-term survival (laparoscopy versus laparotomy: for DFS, aHR=1.06, 95% CI=0.64-1.75, *P*=0.826; for OS, aHR=1.10, 95% CI=0.72-1.68, *P*=0.671).

The Cox proportional hazards regression model also showed that for apparent early-stage UCCC, age at diagnosis (≥ 65 years versus < 65 years: for DFS, aHR=1.51, 95% CI=1.07-2.24, *P*=0.003; for OS, aHR=1.39, 95% CI=1.14-2.51, *P*=0.026), the preoperative ASA physical status score (III versus I/II: for DFS, aHR=1.98, 95% CI=1.14-3.27, *P*=0.021; for OS, aHR=2.02, 95% CI=1.10-3.09, *P*=0.016), the 2009 FIGO stage of the disease (III/IV versus I/II: for DFS, aHR=6.98, 95% CI=3.57-13.12, *P*=0.000; for OS, aHR=6.76, 95% CI=2.49-10.68, *P*=0.000), LVSI (Yes versus No: for DFS, aHR=2.14, 95% CI=1.11-2.57, *P*=0.010; for OS, aHR=2.09, 95% CI=1.27-2.92, *P*=0.001), and postoperative adjuvant therapy (chemotherapy/radiotherapy versus No: for DFS, aHR=0.64, 95% CI=0.28-0.97, *P*=0.012; for OS, aHR=0.67, 95% CI=0.32-0.89, *P*=0.033; combined chemotherapy and radiotherapy versus No: for DFS, aHR=0.49, 95% CI=0.23-0.78, *P*=0.018; for OS, aHR=0.55, 95% CI=0.27-0.90, *P*=0.025) were independently associated with the survival.

**Table 4** shows the results of the multivariate Cox proportional hazards regression analyses.

**Table 4:** Multivariate analyses of survival for apparent early-stage uterine clear cell carcinoma.

	DFS			OS		
	aHR	95% CI	*P*	aHR	95% CI	*P*
Age at diagnosis						
< 65 years	1			1		
≥ 65 years	1.51	1.07-2.24	0.003	1.39	1.14-2.51	0.026
BMI at diagnosis						
< 24 kg/m^2^	1			1		
≥ 24 kg/m^2^	1.72	0.91-3.83	0.077	1.67	0.88-3.55	0.109
ASA physical status score						
I/II	1			1		
III	1.98	1.14-3.27	0.021	2.02	1.10-3.09	0.016
2009 FIGO stage						
I/II	1			1		
III/IV	6.98	3.57-13.12	0.000	6.76	2.49-10.68	0.000
Tumor size						
< 4 cm	1			1		
≥ 4 cm	1.40	0.82-2.39	0.218	1.20	0.76-1.91	0.442
LVSI						
No	1			1		
Yes	2.14	1.11-2.57	0.010	2.09	1.27-2.92	0.001
Surgical approach						
Laparotomy	1			1		
Laparoscopy	1.06	0.64-1.75	0.826	1.10	0.72-1.68	0.671
Adjuvant therapy						
No	1			1		
CT or RT	0.64	0.28-0.97	0.012	0.67	0.32-0.89	0.033
CT plus RT	0.49	0.23-0.78	0.018	0.55	0.27-0.90	0.025

aHR, adjusted Hazard Ratio; ASA, American Society of Anesthesiologists; BMI, Body Mass Index; CI, Confidence Interval; CT, chemotherapy; DFS, Disease-free Survival; FIGO, the International Federation of Gynecology and Obstetrics; LVSI, Lymphovascular Space Invasion; OS, Overall Survival; RT, radiotherapy.

## Discussion

By reviewing the data of 254 patients from four Chinese high-volume centers, the current study showed that for apparent early-stage UCCC, when compared with patients who underwent open surgical staging, patients who underwent surgical staging by laparoscopy experienced similar oncological outcomes.

The research topic on the employment of minimally invasive surgery among women with EC is not new. The Gynecologic Oncology Group (GOG) LAP2 study was a prospective randomized controlled clinical study with the purpose to study the feasibility and safety of minimally invasive surgery for clinical early-stage uterine cancer (16, 17). With 2616 patients included, the GOG LAP2 study preliminarily concluded that laparoscopic surgery for clinical early-stage EC was feasible and safe in terms of short-term outcomes and resulted in a lower risk of perioperative complications (16). In 2012, the GOG LAP2 reported its findings regarding oncological outcomes (17). It reported that the 3-year recurrence rates among patients who underwent laparoscopy and patients who underwent open surgery were 11.4% and 10.2%, respectively (17). The difference in recurrence rate by laparoscopy versus laparotomy was 1.14% (90% lower bound, -1.28; 95% upper bound, 4.0) (17). The Laparoscopic Approach to Cancer of the Endometrium (LACE) study, a multinational randomized equivalence study, also reported that for clinical early-stage uterine cancer, the employment of total laparoscopic hysterectomy compared with total open abdominal hysterectomy resulted in equivalent 4.5-year DFS rate (open surgery versus laparoscopic surgery: 81.6% versus 81.0%) and no difference in 4.5-year OS rate (open surgery versus laparoscopic surgery: 92.4% versus 92.0%) (18). Based on the evidence from the GOG LAP2 study, the LACE study, and other studies regarding this topic, minimally invasive surgery is recommended by many clinical practice guidelines as the preferred surgical approach for early-stage EC (5, 19–23).

However, one should note that in the aforementioned studies, the proportion of type II EC (including UCCC) was fairly low (16–18). Due to the rarity, prospectively designed clinical study regarding type II EC is difficult. So far, some retrospectively designed studies about the oncological safety of minimally invasive surgery for type II EC have been published. Including 295 patients from four Chinese teaching hospitals, the study conducted by Xu et al. found that for apparent early-stage uterine serous carcinoma, the approach of surgical staging was not an independent prognostic factor for oncological outcomes (laparoscopy versus open surgery: for DFS, aHR=1.16, 95% CI=0.63-2.12, *P*=0.636; for OS, aHR=1.11, 95% CI=0.52-2.38, *P*=0.794) *(* 24). Comparing DFS between minimally invasive surgery and laparotomic surgery in patients with high-risk EC, the study conducted by Segarra-Vidal et al. included 626 patients (25). Among them, 468 women had type II EC (25). They found that there was no difference in 5-year DFS rate between the open surgery group (53.4%, 95% CI: 45.6%-60.5%) and the laparoscopy group (54.6%, 95% CI: 46.6%-61.8%) (25). They concluded that minimally invasive surgery was not associated with the deterioration of survival among patients with high-risk EC (25). Furthermore, the subgroup analysis showed that the employment of a uterine manipulator during laparoscopy surgery did not worsen the DFS (HR=1.01, 95% CI=0.65-1.58, *P*=0.960), the OS (HR=1.18, 95% CI=0.71-1.96, *P*=0.530), and the recurrence rate (HR=1.12, 95% CI=0.67-1.87, *P*=0.660) among patients with high-risk EC (25). To compare surgical and survival outcomes in patients with early-stage uterine carcinosarcoma managed by laparotomic surgery versus minimally invasive surgery, the study conducted by Corrado et al. included 170 patients and concluded that for women with early-stage uterine carcinosarcoma, there was no difference of oncologic outcome between the two approaches (26). The findings of our study were consistent with that of the aforementioned studies.

Our study also found that some of the classic risk factors that can be applied to predict the prognosis of type I EC were also useful for apparent early-stage UCCC. These risk factors were as follows: age at diagnosis, the preoperative ASA physical status score, the stage of cancer, and the status of LVSI (1, 3, 4, 27–31). However, unlike for low-risk early-stage type I EC, postoperative adjuvant therapy was beneficial to apparent early-stage UCCC (32–35). This was because when compared with patients of clinical early-stage type I EC, patients with clinical early-stage type II EC are at higher risk of extrauterine metastases and disease recurrence (1–4). In our study, nearly one-third of clinical early-stage UCCC patients were eventually confirmed to have extrauterine metastases after surgery. Among them, the most common site of extrauterine metastases was regional lymph nodes. These findings were consistent with that of previously published studies (34–37). The postoperative adjuvant therapy can reduce the risk of disease recurrence among patients of high-risk EC (including UCCC) (33–35).

Our study included 254 patients with apparent early-stage UCCC, this was a large sample in consideration of the rarity of UCCC. Also, almost all included patients in our study underwent guidelines-based management and a long-term follow-up, this can reduce the effect of confounding factors (such as protocol of treatment) on patients’ prognosis as much as possible and enable us to identify the outcomes of interest. However, this study still suffers from some limitations. First, due to the retrospective nature of the study design, this study was at risk of inevitable biases, such as information bias, selection bias, et al. To reduce the possibility of these biases as much as possible, we pre-set inclusion and exclusion criteria and strictly followed them to screen eligible patients, and excluded those cases that lack relevant data. Second, because robotic-assisted minimally invasive surgery for EC is relatively new in these participating institutions, our study failed to explore its impact on oncological outcomes of apparent early-stage UCCC. However, according to the findings of the study conducted by Segarra-Vidal et al, the robotic-assisted minimally invasive surgery was oncological safe as open surgery for type II EC (25). Third, because of the limited resources, the pathological diagnoses of UCCC were not reviewed again by experts in pathology. The last, some variables of clinical significance, such as the protocol and the number of cycles of postoperative adjuvant therapy, comorbidities, etc. were not included in the statistical analysis, mainly due to the difficulty in obtaining these data. This was potentially representing a bias in our analysis.

## Conclusion

In summary, there was no difference in recurrence rate, recurrence pattern, DFS, and the risk of all-cause death when comparing laparoscopic surgery and open surgical staging among women with apparent early-stage UCCC. Although our study has some limitations, the findings of our study support the assertion that surgical staging by laparoscopy did not compromise the survival of women with apparent early-stage UCCC.

## Data availability statement

The raw data supporting the conclusions of this article will be made available by the authors, without undue reservation.

## Ethics statement

Ethical review and approval were not required for the study on human participants in accordance with the local legislation and institutional requirements. Written informed consent for participation was not required for this study in accordance with the national legislation and the institutional requirements.

## Author contributions

Conceptualization: CS, LR, and YT. Methodology: LQ, XG, Hui Xu, CH, L-LZ, and YT. Data collection: all authors. Project administration: CS, LR, and YT. Supervision: YT. Writing - original draft: CS, LR, and YT. Writing - review and editing: all authors. All authors contributed to the article and approved the submitted version.

## Conflict of interest

The authors declare that the research was conducted in the absence of any commercial or financial relationships that could be construed as a potential conflict of interest.

## Publisher’s note

All claims expressed in this article are solely those of the authors and do not necessarily represent those of their affiliated organizations, or those of the publisher, the editors and the reviewers. Any product that may be evaluated in this article, or claim that may be made by its manufacturer, is not guaranteed or endorsed by the publisher.
